# Creutzfeldt–Jakob Disease: Analysis of Four Cases

**DOI:** 10.3389/fneur.2016.00138

**Published:** 2016-08-29

**Authors:** Ali Al Balushi, Marshall W. Meeks, Ghazala Hayat, Jafar Kafaie

**Affiliations:** ^1^Department of Neurology, Saint Louis University School of Medicine, Saint Louis, MO, USA

**Keywords:** Creutzfeldt–Jakob disease, dementia, neuropathology, prion protein, spongiform encephalopathy

## Abstract

**Background:**

Creutzfeldt–Jakob disease (CJD) is a rare, rapidly progressive neurodegenerative disease that almost always results in death in under a year from onset of symptoms. Here, we report four cases of CJD with different clinical presentations diagnosed at our institution over a 2-year period.

**Cases:**

The first patient is an 82-year-old woman who presented with depression, cognitive decline, and word-finding difficulty over 4 weeks. The patient deteriorated neurologically to akinetic mutism and death within 6 weeks of presentation. The second patient is a 54-year-old woman with liver cirrhosis who presented with confusion, ataxia, and multiple falls over 4 weeks. She was treated initially for hepatic encephalopathy but continued to progress to mutism, startle myoclonus, and obtundation. Death occurred within 4 weeks of presentation. The third patient is a 58-year-old woman who presented with an 8-week history of confusion, urinary incontinence, Parkinsonism, ataxia, and myoclonus. Death occurred within 2 months from presentation. The fourth patient is a 67-year-old man who presented with a 6-week history of headache, blurred vision, ataxia, and personality change and progressed to confusion, myoclonus, akinetic mutism, and obtundation. Death occurred within 3 weeks from presentation.

**Conclusion:**

These four cases highlight the varied possible clinical presentations of CJD and demonstrate the importance of considering CJD in patients with atypical presentations of rapidly progressive cognitive decline. To diagnose CJD, brain biopsy remains the gold standard. However, the presence of CSF protein 14-3-3, typical MRI findings and suggestive EEG abnormalities, all support the diagnosis.

## Introduction

Creutzfeldt–Jakob disease (CJD) is a rare neurodegenerative disorder that causes rapidly progressive dementia leading to death. It belongs in a group of diseases known as prion diseases. The central pathological event of CJD is formation of an abnormally folded protein called scrapie prion protein (PrP^Sc^) from the wild-type cellular prion protien PrP^C^. The PrP^Sc^ then acts as a template for more PrP^C^ to be improperly folded into PrP^Sc^ in a process that is poorly understood. Unlike PrP^C^, the PrP^Sc^ is insoluble and cannot be degraded by proteinase enzymes. This substance accumulates and results in the pathological changes characteristic of CJD: spongiform changes of gray matter and neuronal loss without inflammation (1).

The incidence of CJD in the United States is estimated to be 1–1.5 per million per year (2). The age of onset is usually between 55–75 years, median 68 years, and both genders are affected equally. There are four subtypes of CJD: sporadic, familial, iatrogenic, and variant form (3). Sporadic CJD is the most common form of the disease and constitutes about 85–90% of all CJD cases (4). Clinical presentation is manifested by rapidly progressive cognitive decline and varied associated neuropsychiatric manifestations like myoclonus, cerebellar ataxia, visual symptoms, pyramidal and extrapyramidal signs, and akinetic mutism. The median duration of survival is approximately 4.5 months from onset of symptoms, with 90% of patients surviving less than 1 year (4). Unfortunately, CJD is always fatal. Definite diagnosis of CJD requires brain biopsy, which is not always feasible given the highly invasive nature of the procedure. Other limitations of brain biopsy include concerns of transmission to health care workers and reduced diagnostic yield if obtained tissue is insufficient or performed on an area of the brain without spongiform changes/PrP^Sc^ deposition (5–7). These factors can make the diagnosis of CJD challenging, especially if it presents in an atypical way.

Here, we present four cases that we diagnosed with sporadic CJD from June 2014 to March 2016 at St. Louis University Hospital in St. Louis, MO, USA. Table 1 summarizes the cases.

**Table 1:** **Patient characteristics**.

Age/sex	Clinical presentation	Initial diagnosis	Time from onset to diagnosis	MRI findings	EEG findings	CSF 14-3-3	Autopsy
82/F	Memory impairment, aphasia, and depression	Alzheimer’s dementia	7 weeks	Small vessel disease	Generalized slowing	Positive	Positive
54/F	Confusion, ataxia, and falls	Hepatic encephalopathy	6 weeks	DWI and FLAIR basal ganglia hyperintensity	Periodic sharp wave complexes	Positive	Positive
58/F	Confusion, myoclonus, urinary incontinence, ataxia, and Parkinsonism	CJD	10 weeks	DWI and FLAIR hyperintensity in basal ganglia and pulvinar nuclei of the thalami	Periodic sharp wave complexes	Positive	Not done
67/M	Headache, blurred vision, ataxia, personality change, confusion, and myoclonus	Subacute encephalitis	9 weeks	DWI and FLAIR hyperintensity in basal ganglia and left frontal cortex	Periodic sharp wave complexes and slow background	Positive	Positive

## Case Presentations

The first patient is an 82-year-old Caucasian woman who presented with a 4-week history of disorientation, word-finding difficulties, and depressed mood. Prior to the onset of these symptoms, she lived alone and was completely independent. Clinically, she demonstrated fluctuating level of orientation and word-finding difficulties. She deteriorated over the course of 2 weeks and developed apathy, urinary incontinence, and akinetic mutism.

Laboratory tests revealed normal vitamin B12, folate, TSH, ACE level, ESR, ANA, ANCA, TPO antibodies, and complements levels. HIV 1 and 2 and RPR returned negative. A 24-h EEG showed generalized slowing. Brain MRI revealed chronic small vessel ischemic changes. CSF studies demonstrated normal protein, glucose, and cell counts. PET CT scan was done to rule out occult malignancy and was negative for abnormal uptake. CSF paraneoplastic antibodies returned negative.

With more common etiologies of aphasia and rapidly progressing dementia now ruled out, CJD became suspected. CSF 14-3-3 protein and CSF tau protein were both elevated. Brain biopsy was recommended to the family for confirmation of CJD but they declined and instead chose supportive care. The patient passed away 6 weeks later secondary to pneumonia. Autopsy was performed. Her brain was sent to the National Prion Disease Pathology Surveillance Center (NPDPSC) in Cleveland, OH, USA, which confirmed the diagnosis of CJD by detecting PrP^Sc^.

The second patient is a 54-year-old Caucasian woman who presented with increasing confusion, ataxia, and multiple falls. Her past medical history was significant for type 2 diabetes mellitus, non-alcoholic steatohepatitis complicated by liver cirrhosis (Child-Pugh A), and depression. Her symptoms started insidiously over 1 month and then significantly progressed. In the 2 weeks prior to admission, she was not able to walk without support and became mute. On clinical exam, she opened her eyes spontaneously but did not follow commands. She localized pain. Occasional startle myoclonus was also noticed.

Systemic exam showed no evidence of astrexis, ascites, or organomegaly. Lab tests including ammonia, liver enzymes, and INR were within normal range. Blood count and metabolic panel were normal. She was started on lactulose and intravenous thiamine for suspected hepatic encephalopathy. Continued workup including vitamin B12, TSH, ANA, ANCA, caeruloplasmin, TPO antibodies, and ACE level all came normal. HIV 1 and 2 and RPR returned negative. Heavy metal screen was negative. CSF studies including *T. wipplei* and ACE level were negative. Autoimmune paraneoplastic antibodies were negative from both serum and CSF. Brain MRI revealed increased signal intensity in the basal ganglia on DWI and FLAIR sequences.

**Figure 1:**
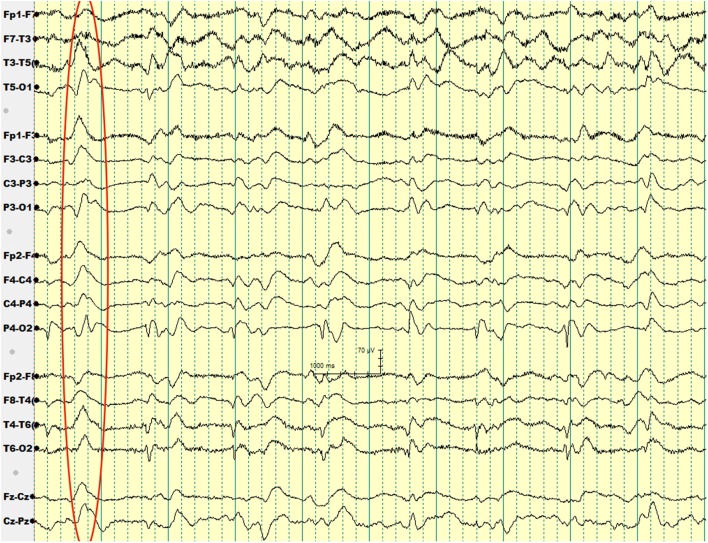
**EEG of patient 2 with periodic sharp wave complexes circled**.

Over the subsequent 2 weeks, her mental status continued to worsen. EEG showed generalized periodic sharp wave complexes and slow background activity (Figure 1). Given her MRI and EEG results, CJD became suspected. Her CSF protein 14-3-3 and CSF tau amount were both elevated. Before we could confirm her diagnosis with a brain biopsy, she developed respiratory failure secondary to aspiration pneumonia and passed away. Autopsy was performed. Her brain was sent to NPDPSC, which detected PrP^Sc^ and confirmed the diagnosis of CJD.

The third patient is a 58-year-old African–American woman who presented with 2-month history of rapidly progressive decline in cognitive function. Two weeks prior to presentation, she developed bladder and bowel incontinence. On exam, she appeared withdrawn and was oriented to herself and place only. She had limb rigidity with bradykinesia and exhibited frequent myoclonic jerks of her upper limbs. Finger to nose test showed mild ataxia.

**Figure 2:**
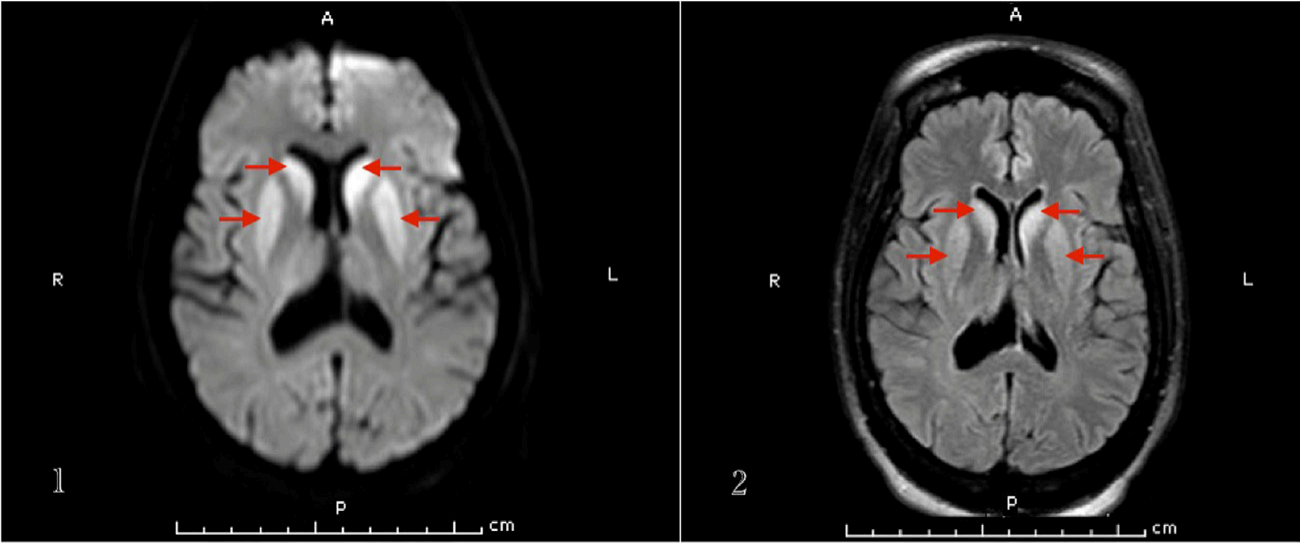
**MRI brain (1. DWI, 2. FLAIR sequence) of patient 3 that demonstrates basal ganglia hyperintense signal**.

Basic labs including TSH, vitamin B12, folate, and ceruloplasmin levels were within normal. HIV 1 & 2 and RPR came negative. Heavy metal and urine toxicology screens were negative. Brain MRI showed increased signal intensity within the caudate nuclei, putamen, and both pulvinar nuclei of the thalami on DWI and FLAIR sequences (Figure 2). EEG showed periodic sharp wave complexes.

CSF studies revealed normal protein, glucose, and cell count. CSF ACE level was normal and CSF VDRL came negative. A therapeutic trail of Carbidopa–Levodopa was tried for the rigidity and bradykinesia, but this did not result in any improvement. Paraneoplastic antibody screen came negative; however, CSF 14-3-3 returned positive. Given strong suspicion of CJD, brain biopsy was advised but patient’s family declined it. She passed away 2 months later. Autopsy was not performed due to family wishes.

The fourth patient is a 67-year-old Caucasian man who presented with a 6-week history of sharp holocranial headache, blurred vision, vertigo, imbalance, and personality change. On exam, he was lethargic, disoriented, and ataxic. His deep tendon reflexes were diffusely exaggerated with bilateral Babinski sign. During his hospitalization, he became irritable, agitated, and aggressive. He subsequently developed startle myoclonus and worsening confusion.

Given his presentation with headaches in addition to mental status change, initial diagnostic consideration included subacute CNS infection. CT head was negative for acute process. Lumbar puncture showed normal CSF cell count and protein. CSF viral, bacterial, and fungal infectious workup was negative. HIV and RPR were negative. Extensive evaluation for encephalopathy yielded; normal metabolic workup, negative systemic infectious process, and negative heavy metal and urine toxicology screens. TSH, vitamin B12, folate, and ceruloplasmin levels were within normal as well as ESR, CRP, and ANA. MRI brain demonstrated mild signal hyperintensity in the basal ganglia and left frontal cortex on DWI and FLAIR sequences. Magnetic resonance angiography and venography were normal. Patient underwent evaluation for occult malignancy with PET CT scan, which did not reveal any focus of abnormal uptake. EEG showed generalized periodic sharp wave complexes and slow background activity. CSF paraneoplastic antibodies came negative. CSF 14-3-3 resulted positive.

The patient’s clinical status deteriorated over the subsequent 3 weeks to obtundation and akinetic mutism. Patient’s family opted for withdrawal of care at this stage. Patient passed away. Autopsy was performed and brain tissue was sent to the NPDPSC, which confirmed the diagnosis of CJD.

## Discussion

This case series demonstrates the myriad of possible presentations of CJD. While our third and fourth patients had a very typical presentation, the first two patients presented with clinical syndromes resembling common alternative diagnoses. In the diagnostic evaluation of a patient suspected of having CJD, it is imperative to first rule out common differential diagnoses, some of which are reversible. These include: vascular, toxic, metabolic, infectious, vitamin deficiencies, iatrogenic, autoimmune, and paraneoplastic etiologies (8). Other neurodegenerative disorders, such as frontotemporal dementia and Alzheimer’s disease (AD) must be distinguished from CJD as well.

The first patient presented with a rapidly progressive cognitive decline and expressive aphasia at the age of 82 years. Given the patient’s advanced age, AD was initially on the patient’s differential diagnosis. However, the rapid progression of her symptoms over only 4 weeks raised concerns about other possible etiologies such as autoimmune encephalitis and CJD. Sporadic CJD typically affects patients aged 55–75. It is rarer in patients over 80 years of age but has been reported in case reports (9, 10).

Alzheimer’s disease can be differentiated clinically from CJD without difficulty, but there are atypical presentations of Alzheimer’s dementia that make this more difficult. Rapidly progressive Alzheimer’s disease has been well described in the literature (11). Van Everbroeck et al. (12) reported the differential diagnoses for 201 patients who underwent evaluation with CSF 14-3-3 for possible CJD, of which 45 patients (22%) had AD as a final diagnosis. In another report from Geschwind et al. (8), 5 out of 67 non-prion diagnoses of 178 patients presenting with rapidly progressive dementia initially suspected of having CJD turned out to be AD. Similarly, in a report from the National Prion Center in Ohio, 352 out of 1,106 brain autopsies performed for evaluation of rapidly progressive dementia were negative for prion disease. Alzheimer’s dementia was diagnosed in 154 patients out of these 352 (43%) (13). Another retrospective study from the Netherlands evaluated brain autopsies of patients with probable or possible CJD over 11 years. This study found only 146 out of 280 patients (52%) had CJD. AD was diagnosed in 40% of the remaining autopsies (14). CJD may present with focal cortical symptoms like aphasia, which is included in the 2007 UCSF criteria for probable CJD (15–17).

Our second patient had a background history of liver cirrhosis and, therefore, her initial presentation was suspected to be hepatic encephalopathy, for which she was treated with lactulose. However, her unresponsiveness to treatment, along with her other neurologic deficits such as ataxia prompted a search for an alternative diagnosis. The patient received an EEG to rule out non-convulsive seizure activity, but it showed periodic sharp wave complexes, a finding that hinted to the diagnosis of sCJD.

Wieser et al. reported that “the EEG in sCJD shows characteristic changes depending on the stage of the disease, ranging from non-specific findings such as diffuse slowing and frontal rhythmic delta activity in early stages to disease-typical periodic sharp wave complexes in middle and late stages to a reactive coma traces or even alpha coma in preterminal EEG recordings” (18).

The diagnostic utility of periodic sharp wave complexes has been demonstrated in multiple studies; Steinhoff et al. (19) found that periodic sharp wave complexes have a high specificity at 91% and a lower sensitivity at 64%. Those figures corresponded to a very high positive predictive value of 95%. Hepatic encephalopathy typically results in EEG findings of generalized slowing and triphasic waves of the frontal lobes (20). However, periodic sharp wave complexes are rather unusual, which helped us to think about the diagnosis of CJD in our second patient.

It can be difficult to distinguish hepatic encephalopathy coexisting with CJD presentation (21), but another hint that helped us make the diagnosis of sCJD in this patient was the brain MRI findings. In sCJD, brain MRI may show signal intensity change in various cortical regions and/or deep nuclei. Zerr et al. found the abnormal pattern of increased signal intensity in the basal ganglia and/or ≥2 cortical areas (temporal, parietal, or occipital) on DWI or FLAIR sequences in 83% of sCJD patients (23). Our second and third patients had typical basal ganglia hyperintensities.

Contrary to the first two patients, our third and fourth patients had a rather typical clinical presentation of CJD and were diagnosed without difficulty. Both presented with a rapidly progressive dementia and suggestive clinical findings of ataxia, startle myoclonus, and akinetic mutism in addition to visual, pyramidal, and extrapyramidal symptoms, characteristic periodic sharp wave complexes on EEG, basal ganglia hyperintensities on brain MRI, and positive CSF 14-3-3, which are all typical findings of CJD. The MRI-CJD Consortium criteria for diagnosis of sCJD are shown in Table 2 (23).

**Table 2:** **MRI-CJD consortium criteria for sporadic Creutzfeldt–Jakob disease**.

**I. Clinical signs**
Dementia Cerebellar or visual Pyramidal or extrapyramidal Akientic mutism
**II. Tests**
Periodic sharp wave complexes in EEG Protein 14-3-3 detection in CSF (in patients with disease duration of less than 2 years) High signal abnormalities in caudate nucleus and putamen or at least two cortical regions (temporal–parietal–occipital) either on DWI or FLAIR sequences

*Probable CJD: two out of I and at least one out of II*.

*Possible CJD: two out of I and duration less than 2 years*.

The median survival time after diagnosis for these four patients was 5.3 weeks, which is less than the expected survival time of 20 weeks (22). This is because the families of most patients opted for palliative care after diagnosis was supported by ancillary studies. Another explanation is possibly our patients presented at relatively more advantaged stage of the disease. Their cognitive decline at onset could have been subtle and went unrecognized until more symptoms became obvious.

## Conclusion

Creutzfeldt–Jakob disease is a fatal neurodegenerative disease that presents with rapidly progressive dementia and a wide range of neuropsychiatric manifestations. CJD usually results in death in less than 1 year after onset. It may present in atypical ways, so it is important for physicians to include it in the differential diagnosis of patients presenting with a rapidly progressive dementia after ruling out common etiologies.

## Author Contributions

AA, JK, and GH were directly involved in the patients’ care. AA wrote the initial draft of the manuscript. MM edited the manuscript and inserted the tables and figures. AA, GH, and JK reviewed and approved the manuscript.

## Conflict of Interest Statement

The authors declare that the research was conducted in the absence of any commercial or financial relationships that could be construed as a potential conflict of interest.
